# Entertainment as Intervention: A Strategic Blueprint for Public Health in Media

**DOI:** 10.7759/cureus.85101

**Published:** 2025-05-30

**Authors:** Shaheen E Lakhan

**Affiliations:** 1 Medical Office, Click Therapeutics, Inc., New York, USA; 2 Department of Neurology, Western University of Health Sciences, Pomona, USA; 3 Department of Neurology, A.T. Still University School of Osteopathic Medicine in Arizona, Mesa, USA; 4 Department of Neurology, Morehouse School of Medicine, Atlanta, USA; 5 Department of Bioscience, Global Neuroscience Initiative Foundation, Miami, USA

**Keywords:** behavior change, entertainment media, health communication, health literacy, health storytelling, influencer marketing, media intervention, narrative persuasion, neuromarketing, public health

## Abstract

Traditional public health messaging struggles to compete for attention in today’s media-saturated environment. This editorial argues for a paradigm shift: embedding life-saving health messages within entertainment and influencer-driven narratives to harness their emotional, cognitive, and behavioral influences. Drawing on neuroscience, behavioral psychology, and media case studies, we demonstrate how scripted content and social media influencers shape public understanding, attitudes, and actions related to health. From increased cancer genetic testing following a primetime television episode to surges in suicide-related searches after controversial dramas, the evidence for narrative impact is robust. We outline a strategic framework for public health professionals to collaborate with media creators at multiple levels of narrative integration, from symbolic embedding to plotline centrality, and propose a sustainable infrastructure to support such partnerships. By treating media not as a risk to mitigate but as a delivery system to optimize, public health can amplify its reach, relevance, and effectiveness.

## Editorial

Over a decade ago, I gave a Grand Rounds lecture at the Cleveland Clinic titled *Hollywood Script: Are Producers the New Doctors?.* The provocation was not rhetorical. At the time, I had just treated a young stroke patient whose family insisted she would fully recover in days, because that’s what they had seen on a popular medical drama. They referenced scenes with poetic voiceovers and dramatic recoveries, expecting the same outcomes. It was a moment of clarity for me. Television and film, the dominant cultural media of our time, were not just background noise; they were shaping how the public understood neurological illness, often in ways that were misleading, romanticized, or flatly incorrect. These portrayals weren’t mere background. They influenced how patients described their symptoms, how families expected recovery trajectories, and even how clinicians justified treatment plans. A seizure as seen on *House M.D.*, for instance, bore little resemblance to actual epileptic activity. Yet it became part of the collective imagination. A stroke recovery, such as the poetic journey in *The Diving Bell and the Butterfly*, moved audiences emotionally but distorted timelines and medical plausibility. Cardiopulmonary resuscitation (CPR) scenes in *ER* suggested outcomes far more hopeful than reality permits. These misrepresentations have clinical consequences.

At the time, I framed the conversation in cautionary terms. Physicians, I argued, must be vigilant and vocal about the ways media distortions impact our field. Today, that framing is no longer sufficient. Caution is not enough. The time has come to shift from reaction to action, from critique to creation. The central thesis of this editorial is simple: if producers have become our de facto public educators, then public health must become their partners.

The public consumes stories not just for entertainment but for orientation. In the absence of reliable, accessible, and relatable health education, people turn to the narratives that populate their screens. These are often fictional and flawed but compelling in their emotional truth. As a result, those truths shape behavior. They influence whether a person gets vaccinated, seeks therapy, completes a screening, or stigmatizes a diagnosis. They affect how communities respond to epidemics, how patients engage with technology, and how entire populations conceptualize health and illness.

I wrote this editorial not just as a physician, but as a neuroscientist, therapeutics developer, and educator. I’ve led the development of software-based treatments that depend on behavior change through neurocognitive and emotional engagement, and I’ve served as a medical advisor to media projects seeking to depict neurological and psychiatric illness responsibly. I have spent decades in the overlapping spaces of medicine, media, and innovation. I’ve seen how powerful a single storyline can be, how it can alter national conversations, catalyze regulatory reform, or even transform clinical expectations at the bedside. I’ve also seen how public health has been slow to claim this domain, often defaulting to public service announcements (PSAs) and print campaigns while misinformation metastasizes across digital networks. We need a new paradigm, one that treats media not as a threat to manage but as a therapeutic environment to optimize.

The screen is no longer a passive surface. It is a neural interface, a conduit into the motivational, emotional, and behavioral systems of the human brain. And if we want to change population health, we must learn to code for that interface. We must learn to write the stories that heal.

The brain power of the story in shaping behavior

The brain is not an information processor in the abstract. It is a pattern-recognition organ tuned to narrative [1]. Humans evolved to understand the world through stories, not statistics. In every culture, from oral traditions to digital feeds, storytelling has served as a vehicle for transmitting values, norms, warnings, and behaviors. This isn’t a literary flourish, it is a neurobiological fact. Story activates regions of the brain far beyond those engaged by raw data.

Narrative transportation, the psychological state of being immersed in a story, is associated with a measurable shift in how the brain processes information [2]. When individuals are “transported” into a story, they exhibit reduced counterarguing, enhanced memory retention, and increased openness to persuasion. Functional MRI studies show that emotional storytelling engages the amygdala, hippocampus, prefrontal cortex, and even motor planning areas, as if the brain is rehearsing the actions portrayed. These networks coordinate emotional salience, memory consolidation, executive decision-making, and even physiological preparation to act. In short, a well-told story is not just heard, it is felt and simulated.

Neuromarketing research has leveraged these principles to shape consumer behavior with extraordinary precision. Brands that associate themselves with characters, emotional arcs, or aspirational identities achieve deeper market penetration because their message is encoded with relevance and emotion. Studies have shown that advertisements embedded within narrative formats (such as branded content or product placement) result in higher recall and more favorable attitudes than traditional commercials [3]. This is not because the message is more informative, but because it is more affectively congruent. The emotional resonance carries the payload.

Public health has been slow to harness this power. Traditional campaigns still lean on statistics, fear-based appeals, or disjointed calls to action. But as countless psychological studies have shown, fear alone rarely changes behavior sustainably. Nor does information. What changes behavior is identity, empathy, and social modeling, all of which are embedded in character-driven narrative. When a beloved character on television grapples with a diagnosis, seeks help, and adapts to life with a condition, that journey becomes a proxy for the viewer’s own contemplation. It offers a script, a role model, and a roadmap.

This neurological pathway is especially important in domains where stigma, misinformation, or ambivalence prevail. Consider mental health, substance use, sexual health, or vaccination. These topics are rarely discussed openly and are often mired in taboo or ideological conflict. Traditional educational campaigns often falter here. But a well-crafted character arc can do what lectures cannot: it can build trust, lower defenses, and spark a change in self-perception. The behavioral scientist BJ Fogg argues that behavior change requires motivation, ability, and a prompt [4]. Narrative delivers all three.

Moreover, storytelling enhances social norming. When multiple characters in a fictional universe validate a behavior, such as discussing trauma, using contraception, or seeking therapy, the viewer perceives that behavior as normative. This perception changes risk appraisal, reduces social cost, and accelerates adoption. Media, therefore, functions not just as an awareness tool but as a behavioral vaccine, one that inoculates against misinformation and equips viewers with scripts for real-life action.

From a neuroscientific perspective, stories rewire the brain. They leave memory traces that shape future interpretation and decision-making. They modulate hormone release, including oxytocin and cortisol, which influence trust and vigilance. They activate the mirror neuron system, creating empathetic resonance that blurs the boundary between observation and experience. All of this makes storytelling a uniquely powerful, and underutilized, public health tool.

To ignore this is to leave one of our most effective instruments of influence on the shelf. If commercial brands can leverage the neuroscience of narrative to increase soda consumption or smartphone usage, public health can use the same tools to increase cancer screenings, mental health engagement, and community resilience. The question is not whether stories affect behavior. The question is: will we write the right ones?

From entertainment to intervention: public health placement in scripted media

Public health professionals and storytellers have long crossed paths through entertainment-education initiatives across the globe, but consistent collaboration and effective implementation at scale remain the exception rather than the norm. The mechanics of public health message dissemination are often grounded in rational persuasion, yet narrative media operate in a different realm altogether. It speaks to feeling, belonging, and identity. This distinction matters profoundly. While facts may inform, only stories inspire. And in a world saturated with competing messages, inspiration is the vector of influence.

To understand how public health can be embedded in entertainment, we must borrow a model that has long been successful in the private sector: product placement. When a soda brand pays for its logo to appear in a hit series, it is not because viewers will immediately buy the drink. It is because viewers subconsciously associate the brand with characters they admire and lifestyles they desire. That same mechanism, affective association, can and must be repurposed for health behaviors.

There are multiple tiers of narrative integration, each with its own complexity and yield (Table 1). Symbolic embedding might involve subtle visual cues: a character wearing a ribbon for breast cancer awareness or using a digital health tool in passing. This low-friction approach preserves artistic integrity while signaling health norms. Behavioral modeling takes this further, showing characters actively engaging with health systems, getting screened, attending therapy, or using assistive devices. These moments, if authentic, normalize real-world behaviors.

**Table 1:** Narrative integration levels for health messaging in scripted media BRCA: breast cancer gene This author-created table outlines a tiered framework for embedding public health messages within scripted entertainment content. The levels range from low-friction symbolic cues to deeply embedded plot-driven health narratives. Each level of integration varies in narrative complexity, audience engagement, and potential for behavior change. Symbolic embedding includes brief visual signals or thematic nods to health norms. Behavioral modeling features characters performing health-related actions, which can normalize and encourage similar behaviors among viewers. Conversational integration occurs when characters engage in dialogue that organically conveys health information, offering relatable scripts for viewers. Plotline centrality places a health condition or decision at the core of a character’s development or a show’s dramatic arc, enabling emotional resonance and deep cognitive processing

Integration level	Definition	Example in practice
Symbolic embedding	Subtle visual or thematic cues signaling a health norm	A character wears a breast cancer ribbon or has a glucose monitor visible
Behavioral modeling	Characters visibly engaging in real-world health behaviors	A protagonist attends therapy, takes medication, or gets screened
Conversational integration	Health topics discussed in natural dialogue, embedded in character interaction	Two friends talk about getting a colonoscopy or managing panic attacks
Plotline centrality	A health issue drives the main story arc or character development	BRCA storyline in *ER*, teen depression, and suicide in *13 Reasons Why*

Conversational integration, where characters discuss health experiences or provide educational dialogue within naturalistic conversation, can blend exposition with character development. When done well, it avoids the dreaded “info dump” and instead becomes emotionally resonant and dramatically functional. The highest level of integration is plotline centrality, where a health issue drives the arc of a story. This requires collaboration between content creators and public health consultants to ensure accuracy without sacrificing narrative tension.

Examples abound, both real and aspirational. A detective series could include a subplot involving opioid addiction and a community-led response, introducing harm reduction principles to a broad audience. A romantic comedy could center on a character navigating infertility, modeling both clinical pathways and emotional realities. A dystopian sci-fi show might incorporate realistic pandemic containment strategies, subtly reinforcing evidence-based public health responses. These are not fantasies, they are opportunities waiting to be claimed.

Importantly, the goal is not didacticism. Audiences do not want to be lectured. They want to see themselves, their fears, and their hopes reflected in characters they care about. Public health messages succeed when they are woven into the emotional fabric of a story, not stitched on like a patch. Integration must be empathetic, character-driven, and dramatically compelling. To achieve this, public health professionals must learn the language of screenwriting, and screenwriters must gain fluency in the science of behavior.

This calls for a new creative alliance, one where medical accuracy is seen as an asset to storytelling, not a constraint. Such partnerships already exist in prototype form. Organizations like Hollywood, Health & Society at USC have facilitated collaborations between health experts and entertainment writers, leading to episodes on major networks that depict HIV prevention, organ donation, and cancer screening with both nuance and accuracy.

But the scale is insufficient. What is needed is an infrastructure that systematizes this partnership model. Just as studios have departments for legal compliance or historical accuracy, they should have health integrity advisors who contribute at the level of story concept, character design, and emotional arc. These advisors should not merely fact-check. They should co-create, ensuring that health content serves both narrative impact and public good.

Public health narratives embedded in entertainment are not a novel experiment. They are part of a long lineage of media interventions that have successfully influenced health behaviors across time, cultures, and platforms. To illustrate their efficacy, we must look at case studies that not only validate the model but also show its range, from broadcast television to global streaming, from Latin American telenovelas to adolescent dramas in the US.

One of the most compelling examples comes from a 2000 episode of the medical drama *ER* [5]. The storyline featured a female character who learns she carries a BRCA1 gene mutation, prompting her to consider prophylactic mastectomy. The episode aired during prime time on a major network and reached millions. Subsequent studies showed that the episode significantly increased public knowledge of genetic risk and testing [5]. More importantly, it led to measurable increases in conversations between patients and healthcare providers and a documented uptick in BRCA testing. The episode didn’t just educate, it activated.

Another landmark case is the telenovela *Simplemente María *(Always Maria), which aired across Latin America in the 1960s. The protagonist, a single mother, learned to sew, pursued literacy, and gained economic independence. The result? A continent-wide surge in adult education enrollment and sewing machine sales. More than that, it changed perceptions of women's autonomy and sparked public discourse on vocational empowerment. The United Nations Educational, Scientific and Cultural Organization (UNESCO) later cited the series as a successful model of entertainment-education [6].

In the US, *13 Reasons Why*, though controversial, undeniably affected public engagement with mental health topics. Its graphic depiction of adolescent suicide sparked global conversations and led to real-time spikes in suicide-related search terms, as documented in *JAMA Internal Medicine *[7]. Schools responded by developing new protocols, and mental health professionals weighed in with both criticism and support. While the show was far from perfect, its impact was unambiguous. The takeaway: when media touches taboo topics with emotional depth, it shapes not only perception but institutional response.

Consider also the example of *Grey’s Anatomy*. Across its long tenure, the show has tackled a range of medical conditions, from organ donation and rare diseases to LGBTQ+ health and reproductive justice [8]. Following specific episodes, national registries noted surges in organ donor signups. Viewer surveys revealed increased empathy and medical literacy. While the series is fictional, its influence is real and measurable.

More recently, the film *Clouds*, distributed on Disney+, told the real-life story of a teenager with terminal cancer. Its compassionate portrayal of palliative care and hospice decision-making led to increased engagement with cancer nonprofits and prompted widespread dialogue about dying with dignity. Viewers were not just moved; they were mobilized, donating, volunteering, and advocating for improved end-of-life options [9].

These case studies are not isolated successes. They are data points in a growing body of evidence that emotionally resonant narratives, when crafted with care and informed by science, can alter behaviors, shift norms, and influence health trajectories. They also reveal the multifaceted nature of public health outcomes. Sometimes the effect is individual: a viewer books a screening or discusses therapy. Other times, it is systemic: institutions change policy, educators revise curricula, and funders recalibrate priorities.

The implications are profound. When scripted content leads to real-world action, when a fictional moment ripples into the public square, we must take note. These stories are not supplements to public health; they are extensions of it. And in a world where algorithms prioritize engagement, emotionally rich, character-driven stories may be the most potent delivery system for public health interventions we have.

The influencer economy: public health in the scroll feed

If television is the theater of health narratives, social media is the street corner. The influencers who command loyalty and trust from millions of followers represent one of the most potent, yet underutilized, avenues for public health communication. The influencer economy is not a sideshow, it is the main stage for digital-native generations. Public health must learn to speak its language (see Table 2 for influence types and public health opportunities).

**Table 2:** Public health messaging opportunities across influencer types This author-created table categorizes common influencer archetypes by the types of public health messages they are best positioned to deliver. Influencers operate within parasocial relationships, offering authenticity and emotional credibility that can enhance message uptake. By aligning public health behaviors with aspirational or identity-reinforcing content, influencers become powerful agents of behavior modeling and norm setting. The examples provided illustrate how health messages can be integrated naturally into existing content formats, increasing reach, cultural relevance, and resonance, especially in underserved or digitally native populations

Influencer type	Public health opportunity	Example in practice
Fitness influencer	Substance use harm reduction, physical activity promotion	Demonstrates naloxone use during Overdose Awareness Week; promotes movement goals
Beauty/lifestyle influencer	Mental health, sexual health, wellness behaviors	Shares therapy journey; shows at-home sexually transmitted infection (STI) test use in a “self-care” routine
Parenting influencer	Pediatric wellness, vaccination, family safety	Narrates child’s doctor visit; discusses childhood vaccines with a trusted tone
Chronic illness influencer	Treatment adherence, navigating care systems	Shows medication routines, insurance navigation tips, and symptom tracking tools

Influencers operate at the intersection of credibility, relatability, and immediacy. Unlike traditional media figures, influencers often cultivate parasocial relationships with their followers, offering glimpses into daily life that create a sense of intimacy and authenticity. When these figures model behavior, whether it’s using a particular product, adopting a new lifestyle, or managing a health challenge, their audiences take note. And often, they follow suit.

Brands have long understood this dynamic. From energy drinks to skincare routines, the influencer ecosystem has been mobilized to shape consumption patterns at scale. Yet public health has largely remained on the sidelines, participating only during moments of crisis or campaign-specific outreach. This is a missed opportunity. Influencers can do more than endorse health messages; they can embody them.

Consider a fitness influencer who demonstrates naloxone use during an overdose awareness campaign or a beauty influencer who talks candidly about her experience with anxiety and therapy, normalizing help-seeking behaviors. A lifestyle vlogger might showcase at-home sexually transmitted infection (STI) testing kits or wearables that monitor sleep and stress. These integrations are not merely informative, they are transformative because they frame health behavior as part of an aspirational, lived identity.

The power of this model extends beyond communication. It opens the door to participatory public health. Influencers can co-create campaigns, crowdsource ideas, run polls, and integrate user-generated content into broader behavior change initiatives. This aligns closely with the principles of citizen science and community-based participatory research. The influencer is no longer a broadcaster; they have become a bridge between science and everyday life.

This model also offers unique advantages for equity. Marginalized communities often face barriers to traditional healthcare communication. Influencers who share their identities, whether based on race, gender, sexuality, disability, socioeconomic status, or geography, can tailor messages to resonate with the lived realities of their audiences. This creates a path for culturally responsive communication that scales with both nuance and speed.

To capitalize on this, public health must invest in influencer engagement not as a marketing afterthought but as a primary strategy. This requires infrastructure: databases of trusted creators, guidelines for evidence-based content integration, funding mechanisms to support authentic partnerships, and metrics that track impact beyond likes and shares. The return on investment may not be immediate, but it is exponential.

A successful influencer-public health partnership is built on mutual trust, narrative authenticity, and aligned incentives. It cannot be coercive or purely transactional. The best outcomes emerge when creators are invited into the design process, empowered to adapt messages for their communities, and supported with data, resources, and shared purpose. These are not mouthpieces; they are co-producers of health impact.

Rather than undermining science, the influencer economy can amplify its visibility and uptake if appropriately harnessed. If we want to make public health visible, desirable, and actionable in the twenty-first century, we must meet people where they scroll.

Who pays, and why it works

A common question, and objection, is who will fund the embedding of public health narratives into entertainment and influencer content. Public health has historically relied on government grants, philanthropic donations, and institutional support, whereas entertainment operates in the realm of profit maximization. But this division is no longer tenable. There is both a fiscal rationale and a moral imperative to fund health messaging in the media people consume every day.

Government agencies like the Centers for Disease Control and Prevention (CDC), National Institutes of Health (NIH), and Department of Health and Human Services (HHS) already spend hundreds of millions annually on public education campaigns. These typically take the form of print materials, social media posts, and short-form videos. However, studies have shown that these traditional standalone formats often fail to generate long-term engagement or behavior change [10]. Redirecting a portion of these funds toward embedded content, in television, film, podcasts, and influencer collaborations, would vastly increase reach and resonance.

Philanthropic organizations like the Robert Wood Johnson Foundation, the Bill and Melinda Gates Foundation, and Bloomberg Philanthropies have long funded public health communication and innovation. These entities are increasingly aware that scaling impact requires new delivery vehicles. Supporting media placements not only aligns with their missions but also offers a highly visible form of philanthropic leverage. A single funded storyline could yield more awareness, discussion, and policy ripple effects than many standalone initiatives.

Health insurers and healthcare systems are also natural partners. These stakeholders have a direct financial interest in improving outcomes and reducing preventable utilization. Imagine a major insurer funding a series of mental health narratives embedded in popular teen dramas to reduce stigma and increase therapy uptake, not as charity, but as part of a long-term member health strategy. Similarly, accountable care organizations and value-based care providers could integrate media into their patient engagement pipelines.

Tech platforms like YouTube, TikTok, Netflix, and Meta also stand to gain. By enabling or even co-producing health-positive content, they improve platform legitimacy, increase viewer trust, and contribute to social impact metrics, all of which are now part of environmental, social, and governance (ESG) assessments that matter to investors and regulators.

The private entertainment industry may resist at first, viewing public health content as a constraint on creative freedom or a drag on commercial appeal, but precedent suggests otherwise. Shows that have integrated social issues, from racial justice and climate change to medical ethics, have often been rewarded with critical acclaim, loyal fan bases, and even award recognition. If done well, health integration adds depth, not didacticism.

Moreover, public health funders can make participation easy. This includes covering the costs of expert consultation, supporting content development with grants or fellowships, underwriting distribution, or providing data analytics support to track engagement and behavior change. These services make the partnership attractive, not burdensome.

In short, multiple actors have aligned incentives to support this model: governments seeking better health return on investment (ROI), philanthropies pursuing social impact, payers targeting behavioral risk, platforms seeking legitimacy, and creatives hungry for meaningful stories. What’s missing is the coordination mechanism, the connective tissue that brings these stakeholders together with clear goals, roles, and shared metrics.

The next step, therefore, is to build the infrastructure to operationalize this vision. Without it, we remain in the realm of pilot projects and missed potential. With it, we can turn storytelling into a full-fledged delivery system for population health.

Building the infrastructure for public health placement

Operationalizing the integration of public health into entertainment and influencer content requires more than good intentions. It demands robust infrastructure, sustained partnerships, and a clear delineation of roles, incentives, and feedback loops. This is not a one-off endeavor, but the construction of an ecosystem that enables continuous, collaborative, and measurable storytelling for health (Table 3).

**Table 3:** Infrastructure components required for narrative-based public health strategy CME: continuing medical education; CDC: Centers for Disease Control and Prevention; NIH: National Institutes of Health; HHS: US Department of Health and Human Services; ESG: environmental, social, and governance This autor-created table outlines the foundational infrastructure elements necessary to scale public health messaging across entertainment and social media ecosystems. Each component plays a distinct role in transforming isolated efforts into a coordinated strategy, ranging from creative production and workforce development to measurement, financing, and regulatory support. Institutional examples highlight the feasibility of implementation across sectors, including government, academic, and philanthropic organizations

Infrastructure element	Primary function	Example of institutional lead or model
Public health media labs	Co-create, prototype, and evaluate health-infused content	Academic centers, public health schools, and nonprofit media institutes
Workforce development	Train storytellers and scientists in cross-disciplinary narrative health	Joint-degree programs, fellowships, and CME offerings
Narrative health consultant	Embed medical-behavioral insight in content development pipelines	Hired roles in production teams; expanded the function of medical advisors
Placement fund	Finance expert involvement and content integration	Multisector funders (government, philanthropy, healthcare payers)
Measurement infrastructure	Evaluate reach, impact, and downstream health behaviors	Research consortia, digital analytics platforms, and health policy think tanks
Policy alignment	Enable funding, regulatory clarity, and programmatic recognition	CDC, NIH, HHS, ESG metrics, public health accreditation bodies

The first element is the creation of public health media labs, interdisciplinary hubs where health experts, writers, producers, technologists, and behavior scientists can co-create content. These labs could be housed within academic medical centers, public health schools, or nonprofit media institutes. Their role would be threefold: to provide consultation to media projects, to prototype content with embedded health goals, and to generate evaluative research on media impact. These labs would serve as engines of both innovation and quality control.

The second element is workforce development. We need a new cadre of professionals fluent in both health science and narrative craft. This includes not only physicians and scientists trained in storytelling but also screenwriters, showrunners, and influencers trained in evidence-based messaging and health ethics. Fellowship programs, joint degree pathways, and continuing education initiatives could cultivate this emerging field of narrative health integration. Embedding narrative skills and media literacy training into public health and medical school curricula can introduce these concepts early, expanding career pathways and equipping future healthcare professionals to participate in narrative-based health interventions from the outset.

Third, we must formalize the role of the narrative health consultant, a professional who participates in writers’ rooms, production cycles, and editorial meetings to ensure that health-related content is both dramatically compelling and medically sound. This role is distinct from traditional medical advisors. It is not about correcting technical jargon; it is about shaping the arc of a story to reflect behavioral science, emotional realism, and social complexity. These consultants should be credentialed, supported, and embedded from the beginning of content development.

Fourth, a public health placement fund should be established to support and subsidize health-oriented content integration. This fund could be managed by a cross-sector coalition and used to finance expert involvement, compensate creatives for health-accurate adaptations, and cover the costs of data collection and evaluation. Grants could be tiered by level of integration, from symbolic embedding to plotline centrality, and awarded based on both narrative potential and expected health impact.

Fifth, we need measurement infrastructure that captures the full spectrum of media effects. This includes traditional metrics like reach and engagement but also newer indicators: viewer sentiment, behavioral intentions, digital search behavior, and downstream actions such as help-seeking, screening uptake, or treatment adherence. Mixed-method evaluation, using both quantitative analytics and qualitative ethnography, will be essential to map the causal pathways between story exposure and health outcome.

Finally, we must foster a policy environment that values and funds media-based public health interventions. This includes integrating media impact metrics into public health reporting, allowing media partnerships to count toward performance benchmarks, and offering regulatory guidance for content creators who wish to incorporate health messages without triggering compliance hurdles.

This infrastructure is not speculative. Each of its components has a precedent. What is novel is the vision of integrating them into a unified architecture of action. Such a system would professionalize the field of public health storytelling, reduce duplication, and accelerate the diffusion of high-impact narratives. It would make it easier for creators to do the right thing and for health leaders to engage culture at scale.

Caveats and ethical considerations

While embedding public health messages into entertainment and influencer content holds transformative promise, it also carries inherent risks. Poorly executed portrayals may oversimplify complex conditions, perpetuate stereotypes, or inadvertently heighten stigma and distress. In my own practice, I’ve seen patients shaped by emotionally charged media depictions that inspired hope but also led to disillusionment when reality diverged from the screen. These effects are not benign. The persuasive power of narrative requires ethical stewardship. Much like clinical interventions, narrative interventions must be evaluated for both efficacy and harm. We must recognize that stories can misinform just as powerfully as they can heal.

To safeguard against unintended consequences, health-media partnerships must center transparency, cultural humility, and rigorous evaluation. This includes community consultation, diverse representation in writers’ rooms, and built-in feedback loops with both audiences and experts. Narrative media is not a replacement for clinical care or public infrastructure, but it can be a complementary force for good if created and governed responsibly. This is not about censorship or creative constraint. It is about co-creating stories that are as emotionally rich as they are behaviorally wise.

Conclusion

The question is no longer whether we can embed public health messages into entertainment media. We can. The question is: why haven’t we? From the binge-worthy drama to the 30-second reel, these narratives shape belief, behavior, and ultimately health.

Just as Coca-Cola pays to appear on a sitcom kitchen table, public health must invest to place an overdose reversal, a vaccine decision, or a mental health tool into a storyline. In the attention economy, the difference between myth and medicine is screen time. The public no longer distinguishes between entertainment and education, and neither should we.

For me, this is not an abstract proposal, it is a professional mandate born of personal urgency. As a neurologist, neuropsychiatrist, and neuroscientist, I’ve dedicated my life to healing brain disease. I’ve also watched as families, patients, and even clinicians have been misled by dramatic depictions and delayed care or lost hope because of them. But I’ve also seen how one well-told story can empower, reframe, and even save a life. That is the power of narrative. It is time to script survival into the stories we tell and to do so with strategy, science, and scale.
